# Fat mass and fat‐free mass in relation to cardiometabolic diseases: a two‐sample Mendelian randomization study

**DOI:** 10.1111/joim.13078

**Published:** 2020-05-04

**Authors:** S. C. Larsson, S. Burgess

**Affiliations:** ^1^ Unit of Cardiovascular and Nutritional Epidemiology Institute of Environmental Medicine Karolinska Institutet Stockholm Sweden; ^2^ Department of Surgical Sciences Uppsala University Uppsala Sweden; ^3^ Department of Public Health and Primary Care University of Cambridge Cambridge UK; ^4^ MRC Biostatistics Unit University of Cambridge Cambridge UK

Dear Sir,

High body mass index is causally associated with most cardiovascular diseases but not stroke [1]. Body mass index is determined by the content of both fat mass and fat‐free mass, which may have opposite effects on cardiometabolic diseases. A Mendelian randomization (MR) study that utilized 82 single‐nucleotide polymorphisms (SNPs) associated with fat mass or fat‐free mass index revealed that fat mass index but not fat‐free mass index was associated with an increased risk of cardiovascular disease amongst 367 703 UK Biobank participants [1]. That study had low power to assess the role of fat‐free mass index and did not assess the associations of fat mass and fat‐free mass with specific ischaemic stroke subtypes or type 2 diabetes. Here, we conduct an updated two‐sample MR analysis of fat mass and fat‐free mass indices instrumented by a large number of SNPs in relation to major cardiometabolic diseases using data from large‐scale genome‐wide association studies consortia [2, 3, 4, 5].

SNPs associated with bioelectrical impedance measured fat mass and fat‐free mass were obtained from analyses by Neale Lab through the MR‐Base platform (http://www.mrbase.org/). Of the 290 and 418 independent SNPs associated with fat mass and fat‐free mass, respectively, at genome‐wide significance (*P *< 5 × 10^‐8^) in up to 331 291 European‐descent individuals of UK Biobank, 581 SNPs were uncorrelated (linkage disequilibrium *R* ^2^ < 0.01 and used as instrumental variables. Clumping of correlated variants was conducted using the TwoSampleMR package in R. We calculated fat mass and fat‐free mass indices by dividing fat mass and fat‐free mass by height squared. Summary association estimates for the cardiometabolic diseases were available from the Coronary ARtery DIsease Genome‐wide Replication and Meta‐analysis plus The Coronary Artery Disease Genetics consortium [2], HERMES consortium [3], MEGASTROKE consortium [4] and the DIAGRAM consortium [5]. This study was approved by the Swedish Ethical Review Authority.

Associations of genetically predicted fat mass and fat‐free mass indices with the outcomes were estimated using multivariable MR analysis [6] conducted with the MendelianRandomization package [7] in R. Results were scaled per 1 kg/m^2^ increase of fat mass and fat‐free mass indices and shown in the Fig. 1. Genetically predicted fat mass index was most strongly positively associated with type 2 diabetes, followed by heart failure, large artery stroke, coronary artery disease, small vessel stroke, ischaemic stroke and any stroke. Genetically predicted fat‐free mass index was not robustly associated with any of the outcomes studied, although the association with type 2 diabetes was in the positive direction (*P* = 0.058).

**Fig. 1:**
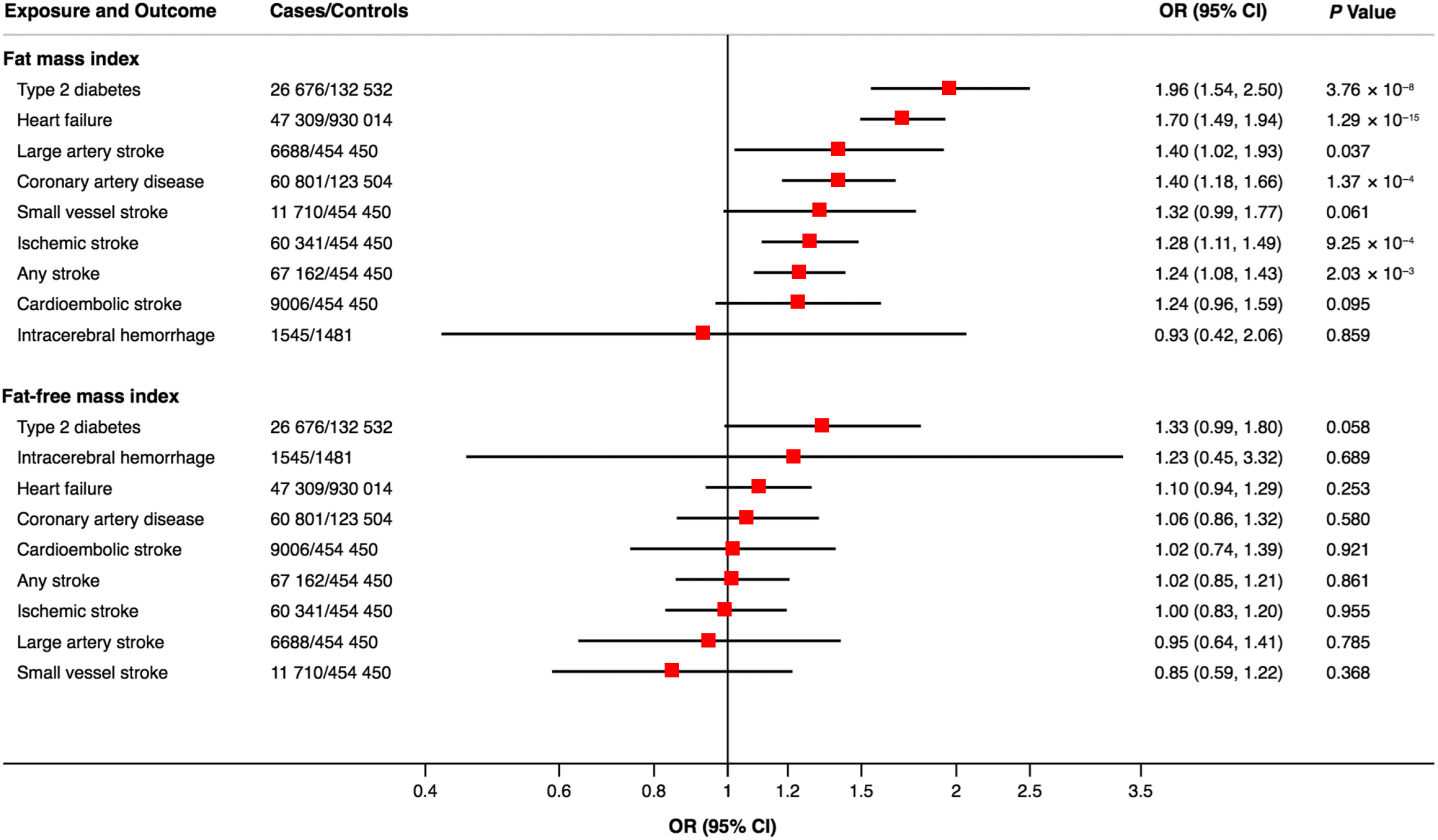
Associations of genetically predicted fat mass and fat‐free mass indices with cardiometabolic diseases. Estimates for fat mass index are adjusted for fat‐free mass index and vice versa.

These findings confirm the results of our previous one‐sample MR analysis of fat mass and fat‐free mass indices instrumented by 82 SNPs in relation to cardiovascular diseases in UK Biobank [1]. Here, we further showed that a high fat mass index is strongly causally associated with risk of type 2 diabetes. Available evidence indicates that body mass index is not associated with total ischaemic stroke or intracerebral haemorrhage [1]. However, the current results provide evidence that increased fat mass has a causal role in the development of large artery and small vessel stroke.

Strengths of this MR study are the large sample sizes for both the exposures and outcomes and the use of multiple SNPs associated with fat mass and fat‐free mass. A shortcoming is that there was partial participant overlap between the exposure and heart failure data sets. This may have produced some bias in the estimates for heart failure in the direction of the observational association. Another limitation is that bioelectrical impedance‐derived measures of body composition can be affected by medical conditions, such as hydration and oedema, and some other factors.

In conclusion, this MR study provides support that higher fat mass index is causally associated with increased risk of major cardiovascular events and type 2 diabetes. Fat‐free mass index appears to have a neutral effect on the studied cardiometabolic diseases.

## Author contribution

**Susanna C Larsson:** Conceptualization (equal); Data curation (equal); Formal analysis (equal); Funding acquisition (equal); Investigation (equal); Methodology (equal); Project administration (equal); Validation (lead); Visualization (lead); Writing‐original draft (lead). **Stephen Burgess:** Data curation (equal); Investigation (equal); Methodology (equal); Validation (supporting); Writing‐review & editing (equal). 

## Conflict of interest

None of the authors has a conflict of interest to disclose.
